# Marker-Less Motion Capture of Insect Locomotion With Deep Neural Networks Pre-trained on Synthetic Videos

**DOI:** 10.3389/fnbeh.2021.637806

**Published:** 2021-04-22

**Authors:** Ilja Arent, Florian P. Schmidt, Mario Botsch, Volker Dürr

**Affiliations:** ^1^Biological Cybernetics, Faculty of Biology, Bielefeld University, Bielefeld, Germany; ^2^Center for Cognitive Interaction Technology, Bielefeld University, Bielefeld, Germany; ^3^Computer Graphics, TU Dortmund University, Dortmund, Germany

**Keywords:** insect locomotion, machine learning, behavioral analysis, marker-less motion capture, deep neural network, motion tracking

## Abstract

Motion capture of unrestrained moving animals is a major analytic tool in neuroethology and behavioral physiology. At present, several motion capture methodologies have been developed, all of which have particular limitations regarding experimental application. Whereas marker-based motion capture systems are very robust and easily adjusted to suit different setups, tracked species, or body parts, they cannot be applied in experimental situations where markers obstruct the natural behavior (e.g., when tracking delicate, elastic, and/or sensitive body structures). On the other hand, marker-less motion capture systems typically require setup- and animal-specific adjustments, for example by means of tailored image processing, decision heuristics, and/or machine learning of specific sample data. Among the latter, deep-learning approaches have become very popular because of their applicability to virtually any sample of video data. Nevertheless, concise evaluation of their training requirements has rarely been done, particularly with regard to the transfer of trained networks from one application to another. To address this issue, the present study uses insect locomotion as a showcase example for systematic evaluation of variation and augmentation of the training data. For that, we use artificially generated video sequences with known combinations of observed, real animal postures and randomized body position, orientation, and size. Moreover, we evaluate the generalization ability of networks that have been pre-trained on synthetic videos to video recordings of real walking insects, and estimate the benefit in terms of reduced requirement for manual annotation. We show that tracking performance is affected only little by scaling factors ranging from 0.5 to 1.5. As expected from convolutional networks, the translation of the animal has no effect. On the other hand, we show that sufficient variation of rotation in the training data is essential for performance, and make concise suggestions about how much variation is required. Our results on transfer from synthetic to real videos show that pre-training reduces the amount of necessary manual annotation by about 50%.

## Introduction

Several insect species are important study organisms in neuroscience, perhaps particularly so in neuroethology. Accordingly, new methodology for the quantitative analysis of insect behavior through motion capture and pose estimation has received a lot of attention. Owing to the computational limitations in image processing, early approaches relied on marker-based tracking algorithms, using kinematic models to constrain the process of pose estimation, particularly if sampling rates were low (Zakotnik et al., 2004) or if multiple body parts had to be tracked (Petrou and Webb, 2012). Additional offline optimization algorithms have been proposed to determine the most likely movement sequence from a series of pose estimates (e.g., Zakotnik and Dürr, 2005). As video hardware improved and sampling rates increased, marker tracking became reliable without an underlying model (Bender et al., 2010). Similarly, current high-end commercial motion capture systems are based on reliable, multi-view marker identification at high frame rates, so as to allow the processing of labeled 3D marker trajectories. Although these systems were developed originally to capture human movement, they can be adapted to track whole-body kinematics of large insects, too, achieving high accuracy and precision even when tracking unrestrained climbing behaviors (Theunissen and Dürr, 2013; Theunissen et al., 2015). Nevertheless, all marker-based approaches are limited by the necessity to equip the animal with an appropriate set of reflective markers. This is not always possible (e.g., on delicate or sensitive structures, for small species, or at locations where markers restrain movement) and requires additional, accurate measurement of all marker positions relative to the body structures that are to be tracked (e.g., particular joints).

With increasing computational power of current image processing systems and the application of machine learning approaches, a number of marker-less motion-capture and pose estimation systems have been developed. They are based on either advanced machine vision techniques such as 3D photogrammetry (Mündermann et al., 2006; Sellers and Hirasaki, 2014) or artificial intelligence applications of deep neural networks (for recent reviews, see Abbas and Masip, 2019; Datta et al., 2019). The latter have been applied very successfully in neuroethology, including insect species as small as *Drosophila melanogaster*. For example, *DeepLabCut* (Mathis et al., 2018) applies a deep architecture of stacked convolutional networks with identity short-cuts, the so-called *ResNet* architecture (He et al., 2016). This *ResNet* part of the system has been trained on the large image data base *ImageNet*. For motion capturing of arbitrary animal movement sequences, *DeepLabCut* appends a stack of de-convolutional layers that can be trained in an end-to-end manner. Other deep neural network applications for motion analysis have focused on particular aspects of this approach, such as iterative improvement by manual re-labeling of pose estimates (Pereira et al., 2019), or exploiting movement information from subsequent frames (Liu et al., 2020). In all of these approaches, the output of the system is a 2D map of probabilities—so-called score maps or confidence maps—that indicate both the most likely position estimate of a particular body part and a measure of confidence of that estimate. With one score map per tracked feature, several features may be tracked in parallel for pose estimation. In fact, training on multiple features in *DeepLabCut* was shown to improve tracking performance over dedicated single-feature trackers (Mathis et al., 2018).

Essentially, the training procedure of deep neural networks is thought to form an internal representation of the feature to be tracked, albeit one of unknown structure and properties. Provided the training data is appropriate, the representation helps to localize a particular instance of the feature regardless of its position, orientation, size, texture or color. However, since the representation is not an explicit geometric model, it is not clear how well it transfers to new applications with setup- or species-specific properties, particularly if these properties have not been part of the training data.

Of course, it is always possible to re-train neural networks to new data sets, but this requires time-consuming, manual annotation. To further improve transfer of neural-network-based motion capture systems to new experimental paradigms, we propose pre-training on synthetically generated video sequences. We argue that this may be particularly suitable for behavioral experiments on arthropods because their exoskeleton and segmented body structure experience little deformation (other than mammals with wobbly masses and relative movement between skin and skeleton). As a consequence, known animal postures may be rendered for arbitrary experimental setups, species-specific body features and animal sizes. Second, manual annotation can be avoided because labels for joints and segments can be generated in conjunction with the generation of each video frame. Third, video frames can be generated in nearly arbitrary sample sizes, allowing for ample feature variation.

The exploitation of tailored synthetic videos to improve transfer learning across experimental paradigms and species appears to be particularly promising in the study of natural locomotion behavior. This is because natural locomotion involves a wide range of manoeuvers such as turning and climbing, the study of which requires the use of very different experimental setups. Furthermore, the analysis of unrestrained locomotion requires reliable and accurate tracking of posture sequences that involve several parts of the body trunk, along with four, six, or even more limbs, each one comprising multiple joints and segments. Finally, animals not only come in different sizes, they also walk or run at variable speed and orientation, generating a lot more postural variation than may be observed in constrained experimental setups.

Accordingly, the main goal of our study is to determine how synthetic video training data may reduce the amount of manual annotation. To this end, a first objective was to find out what kind of and how much variation of geometric transformations is required in the synthetic training data. Aiming at an application to research on unrestrained insect locomotion, our second objective was to demonstrate the efficiency of pre-training on synthetic data in terms of reduced manual annotation of experimental video data.

Our showcase study uses experimental data of walking and climbing stick insects. We will be focusing on stick insects and, in particular, on the Indian stick insect *Carausius morosus* in this study. It is an established organism for studying the neural mechanisms and neuroethology of locomotion (Bässler, 1983; Cruse, 1990; Büschges, 2012; Dürr et al., 2018). In drawing from experimental samples of whole-body postures of *C. morosus* (Theunissen et al., 2014a,b), we generate simplified multi-cylinder models of instant 3D postures, including 22 annotated 3D coordinates of leg, head and thorax joints. Each posture could be transformed by an arbitrary combination of rotation, translation, and scaling. With one posture per frame, we rendered synthetic top-view videos in VGA resolution (640 × 480 pixels) to train *DeepLabCut*.

We show that tracking performance is affected only little by scaling by factors 0.5–1.5. As expected from convolutional networks, the translation of the animal has no effect. On the other hand, we show that sufficient variation of rotation in the training data is essential for performance, and make concise suggestions about how much variation is required. Finally, we assess the transfer performance of the synthetically pre-trained networks on experimental video data from walking stick insects, showing that pre-training reduces the amount of manual annotation by some 50%.

## Materials and Methods

### Video Recordings of Walking Animals

We used intact adult female stick insects of the species *Carausius morosus* (de Sinéty, 1901) from an insect culture bred at Bielefeld University. None of the animals had been used in experiments before. Experimental videos were recorded as top views of animals walking on a planar surface in a “gantry setup.” This setup contained a circular arena with a plane, black surface of 1.2 m diameter. A 200 mm high, dark vertical bar was projected to a circular arena otherwise white arena wall allowed for visual landmark orientation and, thus, induce a directed walking behavior. A digital video camera (Basler A602fc) equipped with a zoom lens (Pentax H6Z810) was mounted on a gantry approximately 1.5 m above the arena. The camera was operated at a resolution of 640 × 480 pixels and a frame rate of 50 frames per second. The gantry allowed to track the animal by moving the camera in two directions, parallel to the walking surface. A total of 13 experimental training videos were recorded: one video for each combination of the four cardinal walking directions and three zoom settings (see Supplmentary Table 1 for estimated intrinsic camera parameters and the corresponding spatial resolution in mm per pixel) with the camera held stationary above the center of the arena. Additionally one extra video was recorded in which the animal was tracked by moving the camera to keep the whole animal in view for the entire video. No further digital processing of videos was done. The intrinsic camera parameters were obtained with the camera calibration functions of OpenCV^1^.

**Figure 1:**
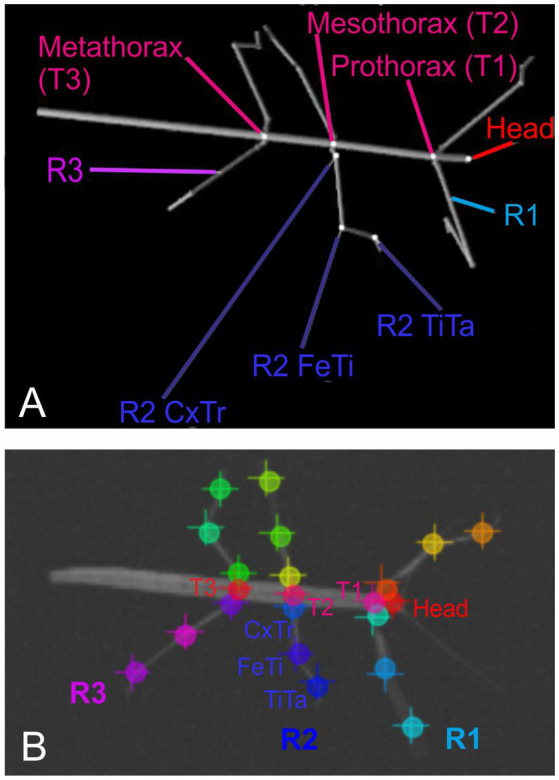
Body geometry of the stick insect and tracked body features. **(A)** Single rendered posture as used for training, with labels indicating the body features that were to be tracked. The right front, middle and hind legs are labeled R1 to R3. The three features per leg to betracked are shown for the middle leg R2: these are the coxa-trochanter joint (R2 CxTr), the femur-tibia joint (R2 FeTi), and the tibia-tarsus joint (R2 TiTa). **(B)** Example frame of an experimental video with labels at the tracked body features. The + symbols mark manually annotated positions; circles mark network estimates. Note that trained networks can deal with motion blur. Despite motion blur of the right front leg and left middle leg, leg postures may be estimated well.

After recording, a set of 286 frames was selected at random and manually annotated using the ImageJ software^2^. On each one of the selected frames, we annotated the positions of 22 body features: These were the coxa-trochanter, femur-tibia, and tibia-tarsus-joints of all six legs, along with the anterior and/or posterior part of the head, prothorax, mesothorax, and metathorax. Apart from the abdomen, this set of features corresponded to the segment boundaries used to generate the synthetic videos (see Figure 1).

### Synthetic Videos

Synthetic videos were generated from rendered body postures acquired in whole-body motion capture experiments as described by Theunissen and Dürr, 2013 and stored in an open-access database (Theunissen et al., 2014a,b). Animated single walking trials are available online^3^ (see samples for the species *Carausius morosus* and obstacle height 0). From this data base, we used joint angle time courses of the first 1,600 frames of the trial named *Animal12_110415_24_33*. For each frame, the 3D locations of 22 body features were calculated, using forward kinematics in *Matlab* (The MathWorks, Natick, MA, USA). The features of the abdomen and the six tarsi of the legs were estimated as described by Dürr and Schilling (2018). Each posture could be subject to scaling, translation and/or rotation so as to control the amount of variation of these parameters in our training data (see below).

**Figure 2:**
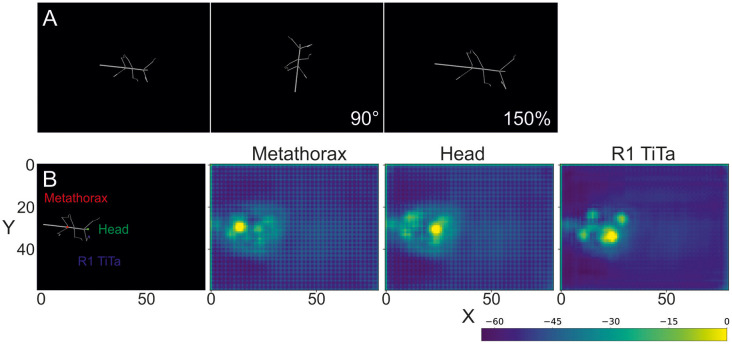
Rendered frames and score maps of a synthetic video. **(A)** Top view of a stick insect whose posture was computed from experimental data, using forward kinematics. The body segments are represented as cylinders and the joints as spheres. As no texture was applied, the animal body has a smooth, gray surface. The above frames show the same posture modified by a rotation (90°) and a scaling by 150%. **(B)** The color labels in the left frame correspond to the score maps of the deep neural network output on the right. The color code shows the confidence score of the network for a particular body feature to be located at that position. In the leftmost case the metathorax position was estimated (red dot in left frame). The middle and right images show score maps for the head (green dot in left frame) and the tibia-tarsus joint of the right front leg (blue dot in left frame), respectively. The axes correspond to the image coordinates in bins of 8 × 8 pixels. The score-map was log-transformed to improve visualization. Since the model outputs probability values in [0, 1], the log transformed values are negative. Warmer colors indicate higher confidence.

Each body segment was visualized as a cylinder with two spheres at the end (Figures 1A, 2A). Individual video frames were generated using Python^4^: individual frames were rendered using the *Vapory* library^5^ and concatenated into video files using the *MoviePy* library^6^. All videos were rendered with an image size of 640 × 480 pixels, i.e., the same as in our experimental videos. Rendering transformed the 3D postures of the body into 2D images of a virtual camera with fixed camera projection matrix. The resulting spatial resolution was 0.28 mm/pixel at a scaling factor of 1, resulting in 0.55 or 0.18 mm/pixel for 50% and 150% scaling. The virtual camera had a viewing angle of 69.4° and was placed 200 units above the ground plane with its line of sight pointing downward, i.e., resulting in a top view video of the walking animal. The surface color as well as the radii for the spheres and cylinders were chosen to approximate the appearance of our experimental videos (for details see Supplementary Material.) As all body features were labeled prior to forward kinematics and rendering, each rendered frame came with 22 labeled feature positions that were used as annotations during training.

The *Matlab* code for calculating the forward kinematics, as well as the *Python* code for rendering images (and videos) and generating the corresponding training annotations are available on request from the corresponding author.

### Deep Neural Network Models

We used *DeepLabCu*t (Deeplabcut github repository^7^, 2018) to train and test all deep neural network models of our study. All training was carried out at the compute cluster of center for Cognitive Interaction Technology (CITEC) at Bielefeld University. It provides several GPU cores of the NVidia Tesla (Tesla P-100 and Tesla C2075) and GTX (GTX 1080 Ti) architectures. We used the standard training schedule of *DeepLabCut*. This schedule comprises training for 1,030,000 iterations with varying learning rates, depending on iteration number. For a detailed description of *DeepLabCut* see Mathis et al. (2018) and Nath et al. (2019).

The subsequent evaluation was performed on one GTX 1050 graphics card. To illustrate the output of such a trained deep neural network, Figure 2B shows score-maps for three body features, where the brightest point on the map indicates the most likely location estimate.

### Experiments

#### Experiment I: The Relative Effect of Scaling, Translation and Rotation

The first experiment was designed to assess the degree of invariance regarding scaling, translation and/or rotation of the animal posture. To this end, we generated a total of seven synthetic videos with a length of 1,600 frames each. In each video the animal was placed at the origin of the world coordinate system and aligned along the *x*-axis. The virtual camera position was adjusted to the position of the animal such that it appeared to be held in place at the image center. We then used any combination of the following three basic transformations to render seven videos:

1. Random translation (T) of the animal in the image plane by [−30, 30]px in either direction;
2. Random rotation (R) of the camera around its viewing axis by [0, 2π] radians;
3. Random scaling (S) of all body segment lengths by a factor drawn from the set {0.5, 0.7, 1.0, 1.3, 1.5}.

All transformations were drawn at random for each frame. Apart from the three videos with single types of transformations, further four videos were generated with combinations of two or all three basic transformations:

4. Video 4 combined random translation with rotation (TR).
5. Video 5 combined random translation with scaling (TS).
6. Video 6 combined random rotation with scaling (RS).
7. Video 7 combined random translation, rotation and scaling (TRS).

For each video frame we then calculated the respective 2D-positions of the annotated body features. Of each synthetic video, we used 200 randomly selected frames (and the corresponding, annotated 2D-positions) to train the neural network model, whereas the total 1,600 frames were used to test the model. The performance of each one of the resulting seven models was evaluated by the accuracy of its position estimates of the 22 body features. The benchmark for evaluation was the TRS Video 7 that contained all possible random combinations of transformation. For each one of the 1,600 frames and each body feature, we calculated the Euclidean distance between the position estimate and the ground truth. The mean of this error measure will be referred to as the average pixel error. Frames for which the model provided position estimates with a confidence rating less than 10% were excluded from the subsequent analysis of the body feature concerned. The latter occurred for frames where the respective body feature was occluded or outside the frame. On average, models which were trained on rotations gave about 30 low-confidence estimates (for 22 features × 1,600 test frames), whereas models which were not trained on rotations gave about 700 low-confidence estimates.

#### Experiment II: How Much Rotation Is Required?

The second experiment was designed to determine a suitable range of rotational variation in the training data. To do so, we rendered eight artificial videos with 800 frames each (corresponding to the first 800 frames of animal pose data). Other than in Experiment I, we kept the position of the virtual camera fixed and only varied its rotation in discrete steps, with one fixed rotation for each video. Training videos were then generated with a random selection of 200 frames drawn from different subsets of these eight videos. Each of these was used to train a separate neural network model. Training Video 1 had no transformation applied. For training Videos 2—8, we used an increasing number of discrete rotations of the virtual camera with increments of 45°. As a result, Video 2 had frames rotated by 0 or 45°, Video 3 had frames rotated by 0, 45° or 90°, and so on. As before, performance was evaluated on the TRS Video 7 of Experiment I, i.e., a video that included rotation angles drawn from a continuous set rather than from a discrete set as used for training. The average pixel error was calculated as in Experiment I.

#### Experiment III: Transfer From Synthetic to Real Videos

The third experiment was designed to evaluate the potential of using synthetically generated video material to reduce the amount of manual annotation of experimental video material. For this experiment, we trained two distinct sets of neural networks: A first set of models used the default networks of *DeepLabCut*, i.e., ones that had been pre-trained on *ImageNet* only. This set of models will be referred to as *Experimental-only* models, and will be used to assess training performance on regular experimental video material. A second set of models was pre-trained on 8,000 randomly selected frames from a synthetic video containing any combination of eight rotations in discrete increments of 45° (0°–315°) and five scaling factors (0.5, 0.7, 1.0, 1.3, 1.5). Then, it was trained additionally in exactly the same way as the *Experimental-only* models. This set of models will be referred to as *Synthetic+Experimental* models, and will be used to assess the benefit of pre-training with synthetic video material.

For the training part on experimental data, we used our videos of real walking animals. The amount of training data was varied in four training fractions: 10, 20, 50 and 80%. The training fraction is the fraction of the total 286 experimental video frames that was used to train the models. The remaining frames (i.e., 1-training fraction) were used as test frames to evaluate the performance of the network. Five models were trained for each training fraction, with each set of training frames drawn at random. As in Experiments I and II, the average pixel error was calculated for the position estimates of 22 body features.

## Results

With our overall goal being to improve marker-less motion capture by use of synthetic video material, we expected the following four aspects of the video generation process to be of importance: (i) the availability of a suitable sample of natural animal postures; (ii) the quality of the rendered image; (iii) the correct choice of image view and scaling; and (iv) sufficient combination and variation of geometric transformations of the rendered animal. Owing to the availability of a database on whole-body kinematics of walking and climbing stick insects (Theunissen and Dürr, 2013; Theunissen et al., 2014a,b, 2015) and the relevance of stick insects as a study organism in locomotion research (e.g., Bidaye et al., 2018; Dürr et al., 2018) we decided to use stick insect data to generate synthetic data. Among the three stick insect species modeled by Theunissen et al. (2014b), the Indian stick insect *Carausius morosus* (de Sinéty, 1901) has the most plain body geometry, allowing us to render postures with a fair degree of realism despite the simplicity of a multi-cylinder-model (see Figures 1A, 2A). Finally, given the availability of high-quality and easy-to-use camera calibration toolkits (e.g., the *Matlab* camera calibration toolkit) it is reasonably simple to measure distance, orientation and optical projection properties of arbitrary digital camera setups. Accordingly, we decided to base all of our experimental analyses of this study on top views of the Indian stick insect *C. morosus*, assuming that most if not all results should easily transfer to: (i) other data sets; and (ii) any other single-camera setup. Instead of addressing (iii) the impact of rendering procedures with different degree of realism of the entire video frame, we decided to focus on the body geometry of the animal model. Thus, the original problem was narrowed down to aspect (iv), i.e., the question of which kind of combinations and how much variation of geometric transformations were needed to be contained in a synthetic training video in order to achieve optimal motion capture performance.

### Random Transformations

**Figure 3:**
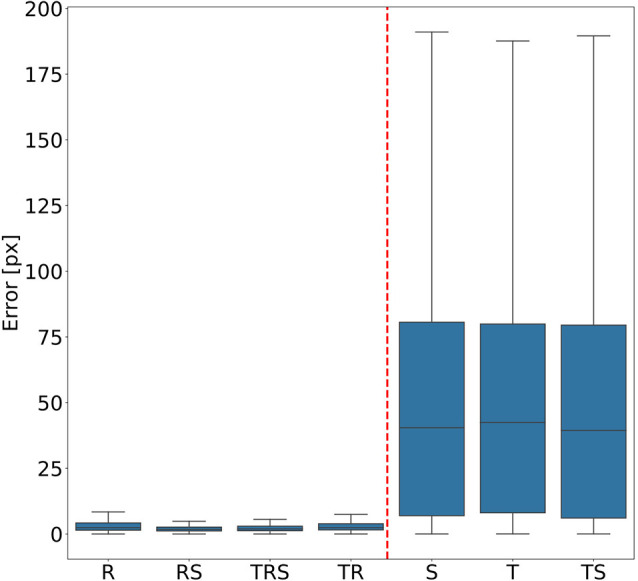
Rotation matters. Average pixel error of seven models that were trained with different combinations of scaling (S), rotation (R), and translation (T). Models that were trained with variable rotation of the posture (left: R, rotation with scaling (RS), translation, rotation and scaling (TRS), TR) outperform models that were trained with one posture orientation only (right: S, T, TS). Note that, for the sake of clarity, outliers beyond 1.5 times the interquartile range are not shown. As yet, all data points were included in any and all statistical computations. The scale factor converting pixels to mm was in the range of 0.18–0.55 mm/pixel, depending on the scaling factor of the random transformation.

In order to judge the relative significance of geometric transformations for motion capture performance, we tested different combinations of linear translation, rotation of the camera and scaling of the body. To do so, a total of seven training videos were generated, using random transformations with parameters drawn from the ranges given in “Experiments” section. The corresponding seven *DeepLabCut* models were evaluated against a test video that comprised all three transformations, again with parameters drawn from the same ranges as the training videos. Figure 3 shows the mean Euclidean distances between the position estimates of the network and the ground truth. Each box plot comprises the errors from all 1,600 frames of the test video and all 22 tracked body features. Clearly, models that were trained on data with variable rotation outperformed the ones without rotation, with the median error dropping from approximately 40 pixels to 3. The highest median error of the models including rotations was 2 pixels and was found for the “R” model, which was trained on rotations only. In metric units, an error of 2 pixels corresponded to 0.36–1.1 mm, depending on the scaling factor of the random transformations. The lowest median error of the models which were trained without variation of rotation was 40 pixels and was found for the “TS” model which was trained on a video with variation of translation only. Furthermore, the small differences among the four models that were trained with variable rotation indicated that models trained with variable scaling performed better than those that were trained without. Translation appeared to have no impact on the performance.

**Figure 4:**
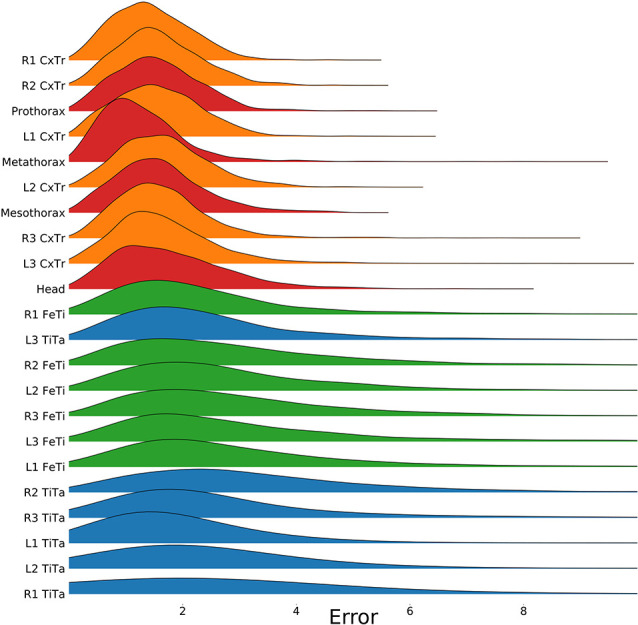
Proximal body parts have lower errors than distal ones. Distributions show the pixel errors for 22 individual body features (*n* = 1,600 per distribution). The error is the Euclidean distance between the ground truth and the network prediction. This particular model has been trained on a video which included random rotations and scaling (RS). Distributions were sorted from top to bottom by increasing standard deviation. The color code groups body parts according to a proximal-to-distal gradient (red: thorax and head; orange: coxae; green: tibiae; blue: tarsi). The scale factor converting pixels to mm was in the range of 0.18–0.55 mm/pixel, depending on the scaling factor of the random transformation.

To test for statistical significance of these observations, we reduced the distributions of error-per frame shown in Figure 3 (*n* = 35,200) to distributions of median errors per body feature (*N* = 22). Since we had corresponding error measures for each one of the seven models, we used a Friedman test to confirm that at least one median value significantly differed from the others (statistic: 119.3; *p* < 0.001). To reveal further performance differences among models we ran *post hoc* pair-wise comparisons using Wilcoxon’s test for matched pairs. The results are shown in Table 1. Performance differences among models proved to be statistically significant for all but four model pairs: These include all comparisons among models trained without variation in rotation (S, T, and TS) and the comparison of the models R and TR. We conclude that models which were trained on different animal sizes in addition to variable rotation showed significantly better performance than models which were only trained with variable rotation and/or translation. Translation, on the other hand, had hardly any impact on performance as variable translation resulted in a slight improvement only if added to variation of rotation and scaling (TRS vs. RS). When comparing the error distributions for different body features, points which are close to the main body axis (e.g., segment borders of the thorax, leg coxae) clearly had smaller errors than those located further away (e.g., femur-tibia joints and tarsi). The example shown in Figure 4 is the result of the RS model, i.e., the network trained with variable rotation and scaling. The color code emphasizes this apparent improvement of tracking performance from distal tarsi (blue), to intermediate femur-tibia joints (green), to proximal groups (red and yellow). The latter were tracked very consistently and with high accuracy and precision. To illustrate the striking improvement whenever the training data varied in rotation, Supplmentary Figure 1 shows the same kind of graph as Figure 4 but for the TS model, i.e., the model trained with variable translation and scaling. Note how the error variance is about one order of magnitude larger than that of the RS model shown in Figure 4, irrespective of body feature. Moreover, the clear proximal-to-distal ordering of error magnitude found for the RS model is lost for the TS model. We conclude that optimal motion capture performance requires sufficient variation of rotation in the training data.

**Table 1:** *p*-values of pairwise Wilcoxon tests for the seven models of experiment I.

	R	RS	S	T	TR	TRS	TS
R	–	***	***	***	0.390	***	***
RS		–	***	***	***	***	***
S			–	0.189	***	***	0.783
T				–	***	***	0.095
TR					–	***	***
TRS						–	***

p-values below 0.001 are indicated by ***. Column and row labels refer to the kind of randomized transformation applied to the training data. R, Rotation only; S, Scaling only; T, Translation only; RS, Rotation and scaling; RT, Rotation and translation; TS, Translation and scaling; TRS, Translation, rotation and scaling.

**Figure 5:**
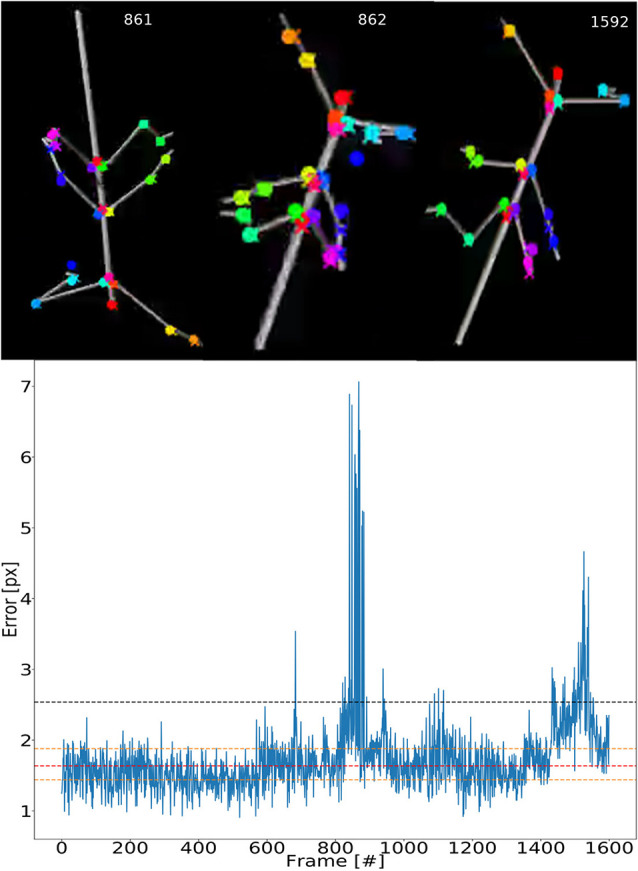
Test error is equally low for most frames. Median pixel errors of all body features were included. The model was trained on video comprising frames with five scaling factors and eight rotations (RS, as in Figure 4). The pixel error varies around a common mean for most video–episodes with three peak regions beyond 3.0 at frames 670, 800–900 and 1,500. These peaks correspond to “difficult postures.” The dashed red line represents the median and the two yellow lines are the first and third quartiles. The black line represents the 1.5 times the interquartile range. The top row shows selected frames from difficult regions. Colored points label the features tracked.

To illustrate the consistency of tracking across an entire synthetic video, Figure 5 shows the error for each frame. The tracking is not uniformly good as there appear to be more “difficult” episodes around frames 850 and 1,500. The top inserts to Figure 5 show some selected frames from these regions. For example, frames 861 and 862 show large tracking errors of the right middle leg tarsus (blue circles). Typically this seems to happen when the tarsus is occluded by the tibia or when the right middle leg crosses the right hind leg. In this case the occlusion also has a detrimental effect on the tracking.

### Significance of Rotation by 180°

**Figure 6:**
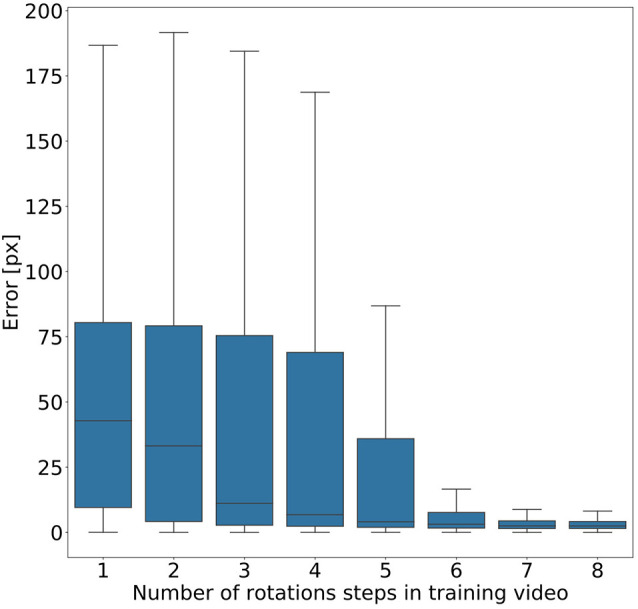
Rotation by at least 180° greatly improves performance. Average pixel error for eight models with varying degree of rotation. Rotations were applied in steps of 45°, with model number indicating the cumulative number of rotation steps. Model 5 is the first model that was trained on postures with a rotation range larger than 180°. Models that were trained on videos with six or more rotation steps have the least error. Note that, for the sake of clarity, outliers are not shown. As yet, all data points were included in any and all statistical computations. The scale factor converting pixels to mm was in the range of 0.18–0.55 mm/pixel, depending on the scaling factor of the random transformation.

Given the conclusion of the previous section, we wanted to find out how much variation of rotation is sufficient. To this end, we ran a second experiment in which eight models were trained on videos that differed in the amount of variation in rotation angles. Instead of random variations, we added one further rotation angle step by step, with increments of 45°. As a result, we obtained eight models, where the first had been trained on animal postures with a single body orientation, the second had been trained on postures with two orientations (0° or 45° rotation), the third had been trained on postures with three orientations (0°, 45°, 90°) and so on. Figure 6 shows that the median error decreased with increasing number of rotation angles added. The error dropped most strongly across the first four models (0°–135° rotation). Furthermore, we found that a major drop in the error range occurs across the first six models (0°–225° rotation) with the addition of the fifth rotation angle, i.e., including 180° rotation (opposite walking directions), marking the steepest decrease in error range. Here the median error dropped from 42 pixels (no rotation) to 3 pixels (corresponding to 0.54–1.65 mm, depending on the scaling factor of the random transformations), and the inter-quartile range of the error dropped from 69 (no rotation) to 37. Given the bilateral symmetry of the animal, the significance of having at least 180° of rotation range suggests that beyond this point it may be easier to tell the front end from the rear end of the animal.

### Transfer to Experimental Data

**Figure 7:**
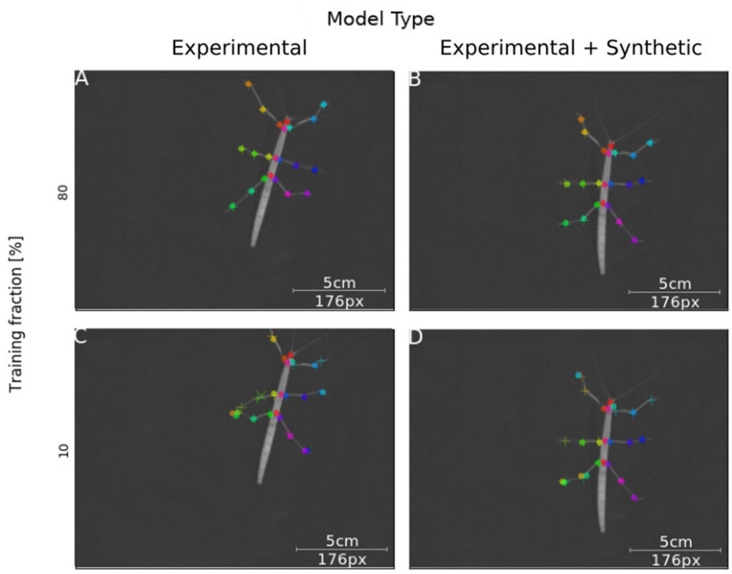
Synthetic training data can lead to good model performance. Selected frames illustrate performance of model variants that do (Synthetic + Experimental, right panels) or do not (Experimental-only, left panels) include artificially generated frames, and differ in the fraction of training data (top panel: 80% of frames were annotated for training; bottom panel: Only 10% of frames were annotated). Circles mark the position of high-confidence estimates of the network, crosses mark low confidence estimates; + symbols mark manually annotated positions. **(A)** The Experimental-only model with a training fraction of 80% achieved good performance (no mis-locations). **(B)** The Synthetic + Experimental model with 80% training fraction achieved similarly good performance as **(A)**. **(C)** The Experimental-only model with 10% training fraction showed some errors and low-confidence estimates. **(D)** The Synthetic + Experimental model with 10% training fraction shows several errors.

Next, we tested how well training on synthetic video data transferred to normal laboratory video material. Real animal video material differs in many ways from our synthetically created videos, for example with regard to noise, motion blur, lens distortion and overall appearance of the animal. Our experimental video comprised 286 manually labeled frames taken from digital videos of a stick insect walking on a horizontal surface in one of eight directions (~45° rotation steps). The image size of the animal was varied by zooming in or out. In this third experiment, a first set of *Experimental-only* models was trained from scratch. For comparison, a set of *Synthetic+Experimental* models was trained using the most advanced model from Experiment I (Figure 3) to start with, i.e., a model trained on postures with variable rotation and scaling. Further, to assess the amount of additional training with manually annotated video material, the fraction of frames used for training was varied in four steps. For example, a training fraction of 20% means that 20% of the 286 annotated video frames were used for training, while the remaining 80% were used for testing. Finally, as the training fraction was drawn at random, a total of 40 models was trained, with five instances per combination of “model type” × “training fraction”. Figure 7 shows representative output examples from four models, two *Experimental-only* models and two *Synthetic+Experimental* models, where each of these pairs (columns in Figure 7) shows one example for a model trained with a training fraction of 80% (top row in Figure 7) and another trained with a training fraction of 10% (bottom row in Figure 7). The results indicate that the performance of both the *Experimental-only* and *Synthetic+Experimental* models are equally good when trained with a training fraction of 80%. The smaller the training fraction, the more frequent become the low-confidence position estimates and mis-location errors.

We assessed whether we could achieve an error margin of ≤4 px and determine how much training data variation was needed to reach this error margin. The 4 px margin was established because on the lower zoom settings this corresponds to about 2.8 mm. As the adult females typically measure about 80 mm (Theunissen et al., 2015), this seemed reasonable. The lower zoom setting was selected as benchmark because here deviations and tracking inaccuracies were larger compared to the size of the insect than on higher zoom settings.

**Figure 8:**
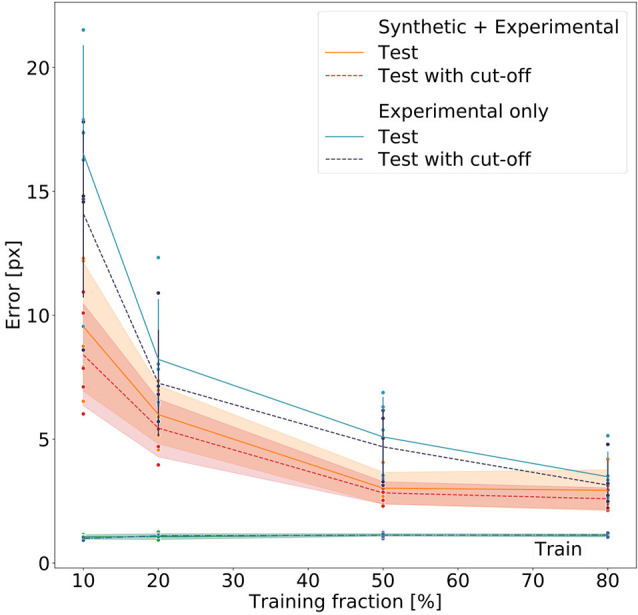
Comparative evaluation of Experimental-only and Synthetic + Experimental models. Individual points represent different networks. Average pixel errors are plotted against the fraction of training frames in the video. Training errors (lower lines) are nearly equal for all networks. Performance of the Synthetic + Experimental models (red and yellow lines with confidence bands) was better than that of models that were trained on experimental data only (blue lines with error bars). Estimates with and without a cut-off threshold of 10 reveal a small improvement if the cut-off is applied, though this difference is not statistically significant. Each dot represents one data point. The scale factor converting pixels to mm was in the range of 0.202–0.771 mm/pixel, depending on the zoom setting of the camera (see Supplementary Table 1).

Figure 8 summarizes the performance for all 40 models (five per model type and training fraction). Both the *Experimental-only* and the *Synthetic+Experimental* models improved with increasing training fraction. Both kinds of model performed similarly well when using a training fraction of 80%, while the *Experimental-only* models performed considerably worse than the *Synthetic+Experimental* models when trained with a training fraction of only 10%. For both model types, training errors were very similar, and generally very low, irrespective of training fraction. Nevertheless median test errors were always significantly lower for *Synthetic+Experimental* models (e.g., Training fraction 10%; Wilcoxon’s *U*-test on rank sums: *U* = 2.0, *n*_1_ = 5, *n*_2_ = 5, *p* = 0.0184), except for the training fraction of 80%. At this training fraction median errors of both the *Experimental-only* and *Synthetic+Experimental* models converged to a similar level of performance and median pixel errors are the same for both *Experimental-only* and *Synthetic+Experimental* models (three pixels, corresponding to 0.6–2.3 mm, depending on the zoom settings of the camera). Only for this highest training fraction did the median error of the *Experimental-only* models fall into the 95% confidence bands of the *Synthetic+Experimental* model errors. Moreover, the confidence cut-off appeared to improve the performance of all models slightly. However, this difference tuned out to be not significant, irrespective of model type (*U* = 169, *n*_1_ = 20, *n*_2_ = 20, *p* = 0.205 for pooled *Experimental-only* models; *U* = 178.5, *n*_1_ = 20, *n*_2_ = 20, *p* = 0.285 for pooled *Synthetic+Experimental* models). Finally, the median error of the *Synthetic+Experimental* models was <3 pixels with a training fraction as small as 50%. We conclude that the use of synthetic videos for pre-training of deep neural networks can reduce the amount of manual annotation by at least 37.5% (3/8) without a decrement in performance.

## Discussion

### Requirements on Feature Variation

While deep neural networks have been repeatedly shown to achieve very good performance in marker-less motion capture (Datta et al., 2019), the choice of appropriate training data remains crucial for their performance. Mathis et al. (2018) showed that accuracy of *DeepLabCut* mainly depends on two factors: the number of frames used for training and the number of body parts to be tracked. They reported that 200 frames are sufficient to reach a good performance level. Figures 3, 6 confirm this. As yet, both of these figures also prove that the amount of parameter variation has a profound impact on performance, particularly the variation of rotation. As expected for a fully convolutional neural network, translation has no impact on the performance of *DeepLabCut*. In contrast, scaling has a small but statistically significant effect (Figure 3 and Table 1), at least within the tested range of 0.5× to 1.5×. It is possible that larger deviations in size will affect the performance more. In experiments on insects, the tested scaling range should cover samples from three to five successive developmental stages. For example, in stick insects of the species *Carausius morosus*, a threefold increase in size would cover the size difference between larval Stages 1 and 3 or between Stages 3 and 7 (assuming size data of Ling Roth, 1916). The scale variation in the experimental data set should exceed this range, synthetic generation of training data would allow for arbitrary size variation during training.

Clearly the main problem of *DeepLabCut* concerns image rotations, which have to be learnt entirely from rotational variation in the training data. Although rotation equivariance has been implemented in neural network applications (e.g., Cohen and Welling, 2016; Chidester et al., 2018), it is not a property of *ResNet* (He et al., 2016) nor of systems that are based on it, including *DeepLabCut*. As a result, if rotational variation in the training data was insufficient, the net will perform poorly during the tracking task if it encounters small deviations in body orientation.

Figure 6 shows that five rotations (0–180°) are sufficient for good generalization in our motion tracking showcase. This makes sense because including the +180° rotation allows the network to tell the rear from the front end of a bilaterally symmetric animal. Together with only three intermediate rotational steps, the system can successfully track animals which are oriented along angles that the network never experienced during training. Data variation beyond 180° rotation further reduced the tracking error, but to a much smaller degree. Although our results suggest that five rotations in steps of 45° are enough to generalise across all rotations, the actual number of rotational steps in the training data may not be crucial. Since the average error dropped markedly only after the network had been trained on the frameset including the 180° rotation, it may be the range that is important, rather than the number of steps comprised in it. At present, we cannot distinguish between these two possibilities.

### Benefits and Limits of Pre-training With Synthetic Data

**Figure 9:**
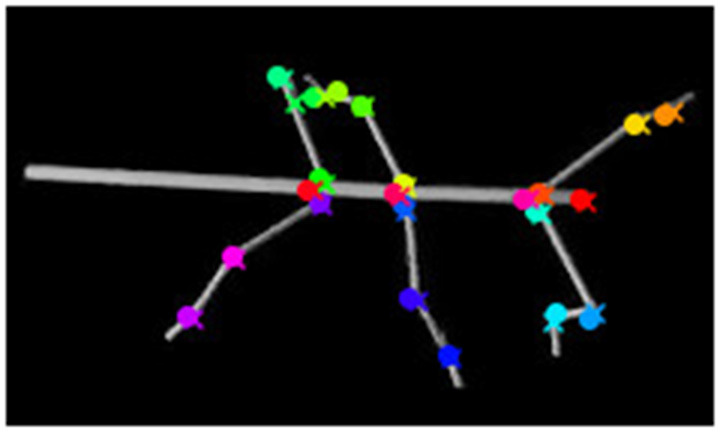
Occlusion of body parts. Here the tarsus of the left hind leg (dark green cross) is occluded by the hind leg femur. The network mistakes the tarsus of the middle leg (light green cross) as the tarsus of the hind leg (dark green circle). The ground truth is marked by crosses, whereas circles mark network estimates.

As shown by Figure 5, tracking accuracy is persistently accurate for long test sequences. The inserts to Figure 5 also show that if performance falls short of the average accuracy, the underlying causes may not be clear. This may be because at least two important properties of the deep neural network approach have both advantages and disadvantages. For example, the ability to learn features of 2D projections of 3D postures is a major strength of deep convolutional networks. As yet this may cause problems if 2D projections involve occlusions (e.g., see Figure 9) because the learnt feature may be masked by a feature of the occluding body part. Thus, if occlusions are frequent, they may either have to be learnt as a separate feature as such, or else be disambiguated by additional camera views. Combining multiple views has been proposed in tracking systems such as *DeepFly3D* (Günel et al., 2019) or *Anipose* (Karashchuk et al., 2020). With multiple camera views, the utility of synthetic video data should be potentiated by the number of cameras, *n*, as each posture of the database is rendered (and automatically annotated) n times, thus reducing the amount of manual labeling by (*n* − 1)×F for F training frames.

Another property that comes with benefits and problems alike is the use of score maps with probability estimates. Whereas this has obvious advantages in case of motion blurring (e.g., see right front leg R1 in Figure 1B), peak confidence values may not always indicate the correct location of a feature. In case of motion blur, a feature may almost vanish but still be identified by means of its most likely position estimate. Occasionally, however, juxtaposed features may lead to score maps with multiple local maxima of the confidence rating. In such cases, the global maximum need not yield the most accurate estimate. We argue that in both of these cases the use of synthetic training data may improve performance on experimental data, for example if motion blur reduces manual annotation accuracy, or if rare “difficult postures” require particularly large training data sets that are easy to synthesise but difficult to obtain through manual annotation.

We had expected the *Synthetic+Experimental* models to transfer features learned from synthetic data to real video frames, such that experimental analysis could be run after additional training with very small sets, e.g., 20–30 video frames. This was not quite the case, despite the fact that *Synthetic+Experimental* models performed significantly better than *Experimental-only* models when trained with low frame numbers (see Figure 8; training fractions of 10–50%). As shown in Figure 7D, a *Synthetic+Experimental* model that was trained with additional 28 experimental video frames shows considerable errors. For the corresponding *Experimental-only* model in Figure 7C this was expected because 28 training frames were far less than the minimal requirement on training data as determined by Mathis et al. (2018).

Nevertheless, Figure 8 clearly shows that pre-training on synthetic data reduces the amount of manually annotated experimental training data by some 50%, thus effectively halving human effort. Despite training errors were equally low for all models, *Synthetic+Experimental* models outperformed all *Experimental-only* models that were trained with <150 frames (e.g., training fraction 50%). Since generating and annotating synthetic frames is done automatically and considerably faster than manual labeling, the reduction of human effort and work time is considerable.

It should be noted that our synthetic data used a rather simplistic multi-cylinder model, suggesting that increased realism of the synthetic data may lead to further improvement. In applications for automated human pose estimation, more complex synthetic data sets have been used (e.g., Varol et al., 2017). Our results show that pose estimation applications on single insect species can achieve good performance with much simpler body shape models. We attribute this to much smaller inter-individual variation of body shape in insects than in humans. Future work will need to test whether and how body shape models have to become more sophisticated if single trained networks were to be applied to multiple species and both sexes. In classification problems involving composite images of multiple, geometrically simple objects, synthetic training images may not need to be rendered at all, but rather be generated by image processing. For example, Toda et al. (2020) successfully applied a technique called domain randomization to create synthetic images of grains with a high degree of variation. While such approaches nicely illustrate the power of modern image processing techniques, it is unlikely that deep learning applications to motion capture and/or pose estimation of animals could be trained successfully on synthetic images generated without an underlying body model.

In summary, we have presented a show-case example of unrestrained walking stick insects, showing that training with synthetic data can effectively reduce the amount of manual data labeling for *DeepLabCut*, a deep convolutional neural network for motion tracking and pose estimation. Provided that the synthetic training data includes sufficient variation of rotation, even a simple multi-cylinder representation of the model animal can reduce the amount of manual annotation by some 50%.

## Data Availability Statement

The raw data supporting the conclusions of this article will be made available by the authors, without undue reservation.

## Author Contributions

MB and VD designed research and advised IA. VD provided motion capture data. IA and FS conceived and wrote the software code. IA conducted experiments and analyzed results. IA, FS, and VD wrote the manuscript. IA, FS, MB, and VD edited and approved the manuscript. All authors contributed to the article and approved the submitted version.

## Conflict of Interest

The authors declare that the research was conducted in the absence of any commercial or financial relationships that could be construed as a potential conflict of interest.

## Footnotes

^1^http://opencv.org

^2^http://imagej.net

^3^http://movement.cit-ec.de/stick-insect-locomotion/animations.php

^4^http://www.python.org

^5^http://github.com/Zulko/vapory

^6^http://github.com/Zulko/moviepy

^7^http://github.com/alexemg/deeplabcut
