# Supplementary material for: Marker-Less Motion Capture of Insect Locomotion With Deep Neural Networks Pre-trained on Synthetic Videos
Source: Front Behav Neurosci. 2021 Apr 22;15:637806. doi: 10.3389/fnbeh.2021.637806 (PMC8100444; doi:10.3389/fnbeh.2021.637806)
Supplement: Supplementary file 2 [file Data_Sheet_1.pdf]

Supplementary Material

for the paper entitled “Marker-less motion capture of insect locomotion with deep neural networks pre-trained on synthetic videos”, by Ilja Arent, Florian P. Schmidt, Mario Botsch, Volker Dürr

Supplementary Table S1: Intrinsic camera parameters and spatial resolution for the four different settings of experimental videos.

Setting	Focal distance [pixels]	Principal point [pixels]	Resolution [mm/pixel]
**Zoomed Out**	(1450.422, 1480.145)	(463.928, 203.846)	0.771
**Zoomed Medium**	(3162.254, 3201.060)	(345.620, 449.841)	0.355
**Zoomed In**	(5597.841, 5595.020)	(315.557, 219.036)	0.202
**Tracking**	(3976.062, 3980.329)	(314.573, 358.758)	0.284

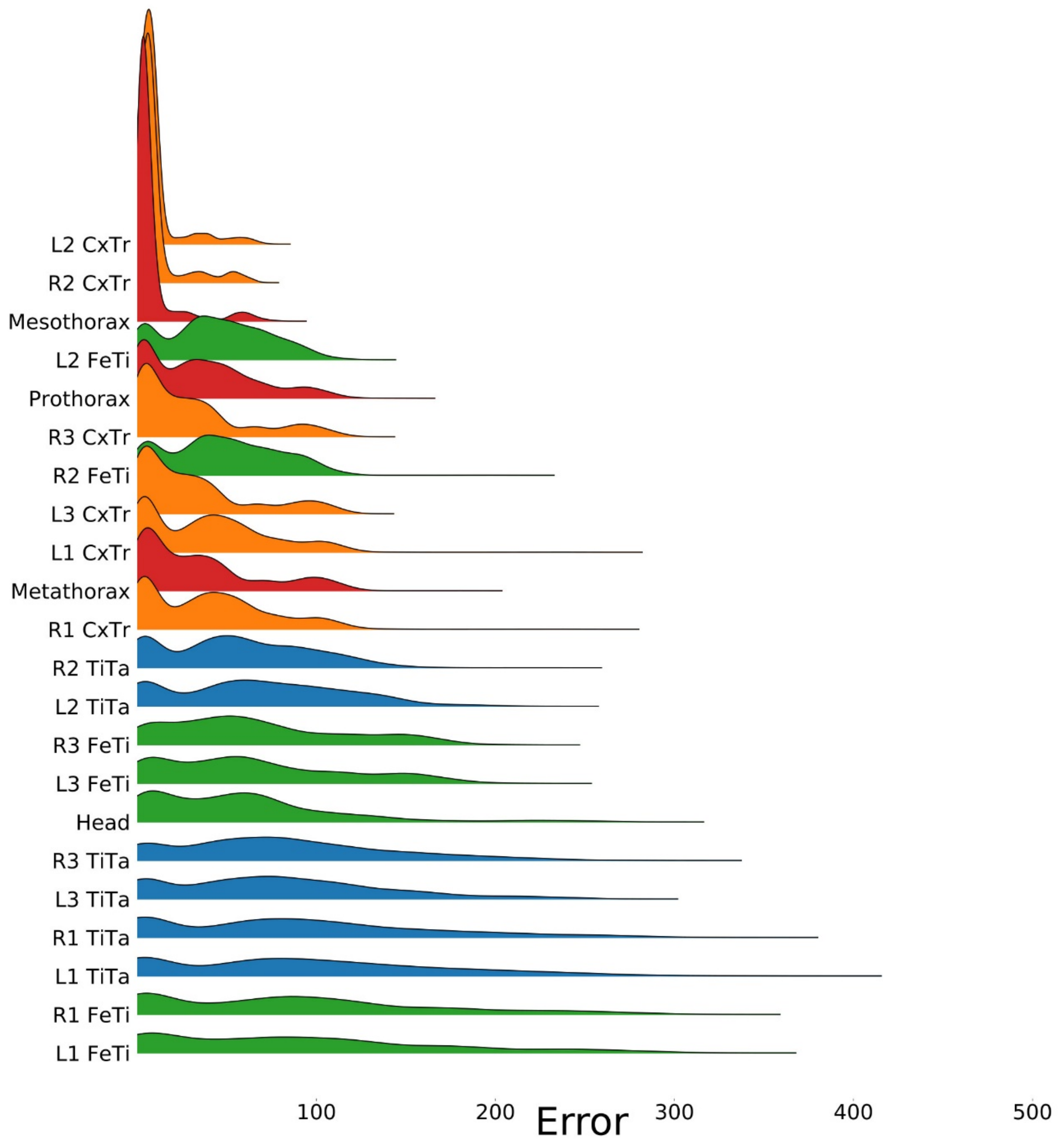

Supplementary Figure S2: Densities of the pixel error for individual body parts. The error is the Euclidean distance between the ground truth and the network estimate. Other than the model used for Fig. 6, this particular model has not been trained on data with varying rotation. Distributions were sorted from top to bottom by increasing standard deviation. As in Fig. 4 the colour code groups body parts according to a proximal-to-distal gradient (red: thorax and head; orange: coxae; green: tibiae; blue: tarsi). In comparison with Fig. 4 errors here are 10 times larger or more. Also, the proximal- to distal gradient of increasing error seen in Fig. 4 is not discernible.
